# Quantitative and qualitative profiles of circulating monocytes may help identifying tuberculosis infection and disease stages

**DOI:** 10.1371/journal.pone.0171358

**Published:** 2017-02-16

**Authors:** Marco Pio La Manna, Valentina Orlando, Francesco Dieli, Paola Di Carlo, Antonio Cascio, Gilda Cuzzi, Fabrizio Palmieri, Delia Goletti, Nadia Caccamo

**Affiliations:** 1 Central Laboratory of Advanced Diagnosis and Biomedical Research(CLADIBIOR), Azienda Universitaria Ospedaliera Policlinico P. Giaccone, Palermo, Italy; 2 Dipartimento di Biopatologia e Biotecnologie Mediche, Università di Palermo, Palermo, Italy; 3 Department of Sciences for Health Promotion and Mother-Child Care “G. D’Alessandro”, University of Palermo, Palermo, Italy; 4 Translational Research Unit, National Institute for Infectious Diseases L. Spallanzani, Rome, Italy; University of Cape Town, SOUTH AFRICA

## Abstract

Tuberculosis (TB) is one of the most important cause of morbidity and death among infectious diseases, and continuous efforts are needed to improve diagnostic tools and therapy. Previous published studies showed that the absolute cells number of monocytes or lymphocytes in peripheral blood or yet the ratio of monocytes to lymphocytes displayed the ability to predict the risk of active TB. In the present study we evaluated the ratio of monocytes to lymphocytes variation and we also analyzed the ex-vivo expression of CD64 on monocytes as tools to identify biomarkers for discriminating TB stages. Significant differences were found when the average ratio of monocytes to lymphocytes of active TB patients was compared with latent TB infection (LTBI) subjects, cured TB and healthy donors (HD). By the receiver operator characteristics (ROC) curve analysis the cut-off value of 0.285, allowed the discrimination of active TB from HD, with a sensitivity of 91.04% and a specificity of 93.55% (95% of confidence interval: 0.92–0.99). The ROC curve analysis comparing TB patients and LTBI groups, led to a sensitivity and the specificity of the assay of 85.07% and 85.71%, respectively (95% of confidence interval: 0.85 to 0.96). The upregulation of CD64 expression on circulating monocytes in active TB patients could represent an additional biomarker for diagnosis of active TB. In conclusion, we found that the ML ratio or monocyte absolute count or phenotypic measures show predictive value for active TB.

## Introduction

Tuberculosis (TB) is one of the most important cause of morbidity and death among the all infectious diseases [1]. New diagnostic tools and therapies are needed. Diagnosis of active TB disease still represents a challenge for the clinical management due to the difficulty related to the detection of *Mycobacterium tuberculosis* (Mtb) in sputum [2]. Moreover, the efficacy of therapy which is evaluated by sputum culture conversion, needs several weeks to get results. Interferon-γ Release Assays (IGRAs) are attractive tests for latent TB infection (LTBI) diagnosis. However they have limitations as they cannot distinguish between subjects with LTBI and active TB disease, and are inadequate for monitoring treatment response[3–5]; moreover they also poorly predict those infected individuals who will progress to active TB[2,6–13]. Therefore, it would be very useful to have a simple and rapid method to screen patients with active TB among the LTBI subjects and to evaluate the anti-microbial therapy success.

The absolute number of monocytes or lymphocytes in peripheral blood or yet the ratio of monocytes to lymphocytes (ML ratio) predict the risk of active TB development in HIV-infected patients co-infected with Mtb or in children born from HIV-infected mothers[14–17]. Moreover, recent data also have highlighted that the increase of the ML ratio is associated with changes of gene transcription in monocytes that may influence their functional anti-mycobacterial profiles. [18–20] It has been described that human CD14^+^monocyte are composed by two subsets based on CD16 expression and relative percentages of CD16^+^ monocytes increase along with TB disease severity [18]. However, whether this unbalance is beneficial or detrimental to host defense remains to be elucidated. Moreover, human monocytes are prone to differentiate towards an anti-inflammatory (M2-like) macrophage activation program during Mtb infection[19].

In this paper we have investigated on the ML ratio in subjects with LTBI and in patients with active TB before and after anti-mycobacterial therapy, to correlate this value with the different conditions of infection/disease. The expression of different surface molecules in circulating monocytes was also evaluated.

## Materials and methods

### Characteristics of the enrolled individuals

A total of 173 individuals were prospectively enrolled as here reported and as described in detail previously [20]: (a) Healthy Donors (HD): 31 individuals tested TST and QFT-IT-negative (9 men, 22 women, median age 37 years); (b) LTBI subjects: 37 individuals (21 men,16 women, median age 43 years) who reported household or equivalent close contacts with smear-positive pulmonary TB patients in the previous 3 months, QFT-IT-positive, with negative chest x-Ray results for active pulmonary lesions and no prior preventive therapy performed; (c) active TB disease: 71 individuals diagnosed with active pulmonary TB (with a positive Mtb culture from sputa or broncholavage, 54 men, 16 women, median range 38 years) who started specific treatment <8 days before enrolment (see **Table 1**);(d) 34 cured TB patients (with a previous microbiological diagnosis, 17 women and 17 men, median age 41). Patients were recruited from the L. Spallanzani National Institute for Infectious diseases, Rome and from the Department of Infectious Disease, University Hospital of Palermo. All patients were treated in accordance with Italian guidelines and received therapy for 6 months. Treatment was successful in all participants, all of whom completed the full course of anti-TB chemotherapy. None of the TB patients had evidence of HIV infection, or was being treated with steroid or other immunosuppressive or anti-tubercular drugs at the time of their first sampling. The study was approved by the Ethical Committee of the L. Spallanzani National Institute for Infectious diseases in Rome (approval number 2/2007), and by the Ethical Committee of the University Hospital in Palermo (approval number 13/2013), where the patients were recruited. Informed consent was signed by all participants. QFT-IT was performed as for manufactures instructions.

**Table 1:** Characteristics of study groups.

	Healthy Donors		LTBI		Active TB		Cured TB		Total	
**Enrolled subjects (%)**	31	*(17*.*92%)*	37	*(21*.*39%)*	71	*(41*.*04%)*	34	*(19*.*65%)*	173	*(100%)*
**Median age**	37		43		38		41		40	* *
**Range**	29–60		21–77		17–82		17–73		17–82	* *
**Male gender (%)**	9	*(29*.*03%)*	22	*(59*.*46%)*	54	*(76*.*06%)*	17	*(50*.*00%)*	102	*(58*,*96%)*
**Origin (%)**										* *
**Western Europe**	31	*(100%)*	24	*(64*.*86%)*	16	*(22*.*54%)*	14	*(41*.*18%)*	85	*(49*.*13%)*
**Eastern Europe**	0	*(0*.*00%)*	4	*(10*.*81%)*	33	*(46*.*48%)*	9	*(26*.*47%)*	46	*(26*.*59%)*
**Asia**	0	*(0*.*00%)*	3	*(8*.*11%)*	11	*(15*.*49%)*	3	*(8*.*82%)*	17	*(9*.*83%)*
**Africa**	0	*(0*.*00%)*	5	*(13*.*51%)*	10	*(14*.*08%)*	4	*(11*.*76%)*	19	*(10*.*98%)*
**South America**	0	*(0*.*0%)*	1	*(2*.*70%)*	1	*(1*.*41%)*	3	*(8*.*82%)*	5	*(2*.*89%)*
**TST (%)**										
**Positive**	0	*(0*.*00%)*	0	*(0*.*00%)*	15	*(21*.*13%)*	0	*(0*.*00%)*	15	*(8*.*67%)*
**Negative**	0	*(0*.*00%)*	0	*(0*.*00%)*	4	*(5*.*63%)*	0	*(0*.*00%)*	4	*(2*.*31%)*
**N.D.**	31	*(100%)*	37	*(100%)*	52	*(73*.*24%)*	34	*(100%)*	154	*(89*.*02%)*
**IGRA TEST (%)**										* *
**Positive**	0	*(0*.*0%)*	37	*(100%)*	49	*(58*.*2%)*	20	* *	106	*(61*.*27%)*
**Negative**	8	*(25*.*8%)*	0	*(0*.*0%)*	9	*(16*.*5%)*	2	* *	19	*(10*.*98%)*
**Indeterminate**	0	*(0*.*0%)*	0	*(0*.*0%)*	2	*(3*.*8%)*	3	* *	5	*(2*.*89%)*
**N.D.**	23	*(74*.*2%)*	0	*(0*.*0%)*	11	*(21*.*5%)*	9	* *	43	*(24*.*86%)*
**TB diagnosis (%)**										* *
**Microbiological diagnosis**					71	*100%*			71	*(41*.*04%)*

### Full differential blood counts

Full blood counts (FBC) of peripheral blood collected in ethylene-diamine tetra-acetic acid (EDTA) containing tubes were performed by one clinical diagnostic laboratory using a five-part differential hematology analyzer(Coulter 4.500, Germany). Full blood count measurement was under strict quality procedures including twice-daily high and low internal quality control, fortnightly quality controls done by the clinical laboratory QC scheme and annual quality assurance as part of clinical laboratory QC scheme. The laboratory is accredited by the Italian National Accreditation System in accordance with international standards ISO17025/2005 and ISO 15189/2007.

### Cell staining and flow cytometry analysis

Peripheral blood was drawn from patients and control donors. Surface staining was performed on freshly drawn heparinized whole blood using mAbs toCD11c, CD16, CD32, CD64, CD70, CD80, CD86, CD123, CD137, CD137-L, CD152, CD163, CD206, HLA-DR, RANK, RANK-L, CCR2, CCR3, CCR4, CCR5, CCR6, CCR7, CXCR2, CXCR3, CXCR5, CD3, CD19, CD56 and their isotype controls, all from BD Bioscience, San Diego, USA).One hundred microliters of blood were incubated with saturating amounts of the mAbs for 20 minutes on ice and then were lysed with FACS Lysing Solution (BD Biosciences). Four-parameter FCM acquisition and analysis were performed on a two-laser FACSCalibur instrument (BD Biosciences) using CellQuest software (BD Biosciences). For a clear analysis, at least 50,000 total events were analyzed. Samples were analyzed with FlowJo software (TreestarInc Ashland, OR). Monocytes were gated first by forward angle and side scatter profiles, followed by gating on the CD14^+^ cell population, and intersecting the two gated populations, for further analysis. Lymphocytes were gated first by forward angle and side scatter profiles, followed by gating on cells expressing CD3, CD19, CD16 and CD56. Standardization of analysis and comparability of results were related to the use of reagents from the same producing company, instrument compensation and gating strategies performed by the same operator at both time points for all subjects studied.

### Statistical analysis

To calculate the ML ratio, the absolute monocyte count was divided by the absolute lymphocyte count. The median or geometric mean was used for descriptive statistics for each parameter. The non-parametric Kruskal-Wallis was performed comparing the medians of ML ratio, significance was settled for *p*<0.0001. The relationship between variables was evaluated using Spearman rank correlation test. A two-side *p*<0.05 was considered statistically significant. Data were analyzed using the Statistic software (Stat soft, Ohio, USA) and GraphPad prism, version 5.0 (GraphPad Software, San Diego, CA,USA). Negative binomial regression model was used to estimate the association between active TB and explanatory variable. To evaluate the performance of each candidate biomarker in discriminating between patients with active disease and LTBI subjects and establish probability cutoff values, logistic regression analysis was performed followed by Receiver operating characteristic (ROC) curve analysis.

## Results

### A high ML ratio is associated with active TB disease

**Fig 1:**
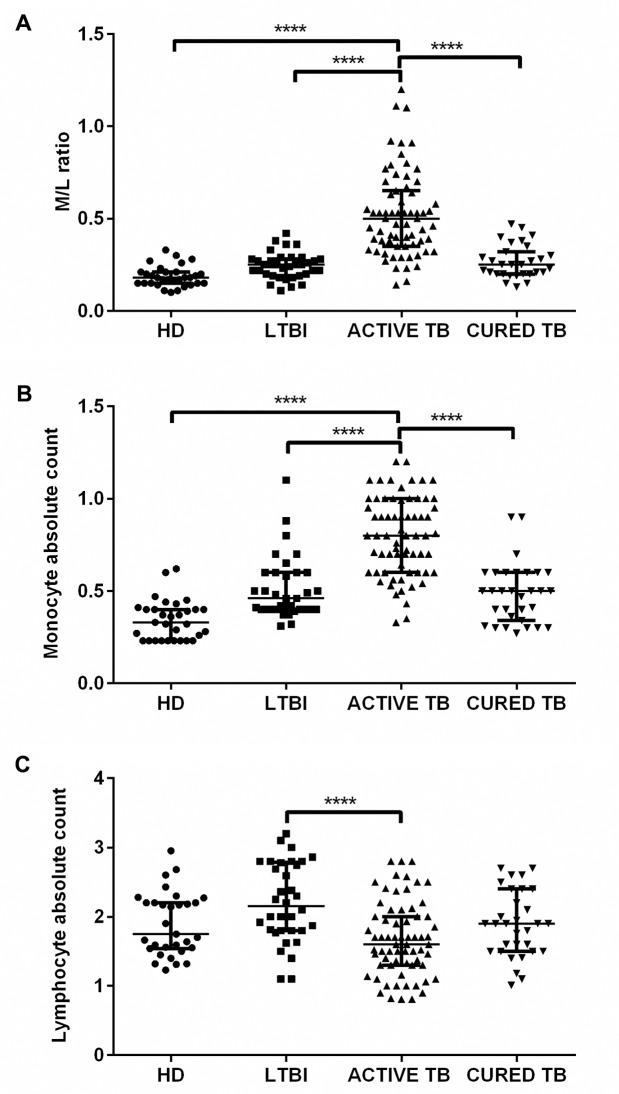
Ratio of monocytes to lymphocytes (ML ratio) of patients with active TB disease, LTBI subjects, cured TB patients and HD. A. Each dot represented ML ratio of a studied individuals. A horizontal bar indicates the median of each group. Significance of differences between groups was compared using Kruskal-Wallis test. *p<0.00001. B. Monocyte absolute count of active TB patients at diagnosis, LTBI subjects, cured TB patients and HD. Each dot representes the value of an individual subject. Each horizontal bar represents the median of each group. *p*<0.00001 were considered statistically significant. C. Lymphocyte absolute count of active TB patients at diagnosis, LTBI subjects, cured TB patients and HD. Each dot represents the value of an individual subject. Each horizontal bar represents the median of each group. *p*<0.00001 were considered statistically significant.

We assessed if ML ratio is modulated by the clinical TB condition. As shown in **Table 2**and **Fig 1A**, patients with active TB disease showed a higher ML ratio (Median:0.50, IQR:0.36–0.64), compared to HD (Median: 0.18, IQR: 0.15–0.21) and LTBI subjects (Median:0.25, IQR:0.20–0.28). Additional analysis showed that the ML ratio value decreased close to the normal range after anti-mycobacterial therapy (Median:0.25, IQR: 0.20–0.31). ML ratio of active TB patients was significantly different compared to that of HD, LTBI subjects and cured TB patients, in all instances with *p*<0.0001. Conversely, no significant differences were observed among HD, LTBI subjects and cured TB patients.

**Table 2:** M/L ratio, monocyte and lymphocyte absolute counts of the subjects enrolled.

TB status		M/L ratio		Monocyte absolute count 1x10^3^/μl		Lymphocyte absolute count 1x10^3^/μl	
	N° of subjects enrolled	Median	IQR	Median	IQR	Median	IQR
**HD**	31	0.18	0.15–0.21	0.33	0.23–0.40	1.75	1.55–2.20
**LTBI**	37	0.25	0.20–0.28	0.46	0.40–0.60	2.15	1.80–2.79
**Active TB**	71	0.50	0.36–0.64	0.8	0.60–1.00	1.65	1.30–2.03
**Cured TB**	34	0.25	0.20–0.31	0.5	0.33–0.60	1.9	1.50–2.18

**Fig 2:**
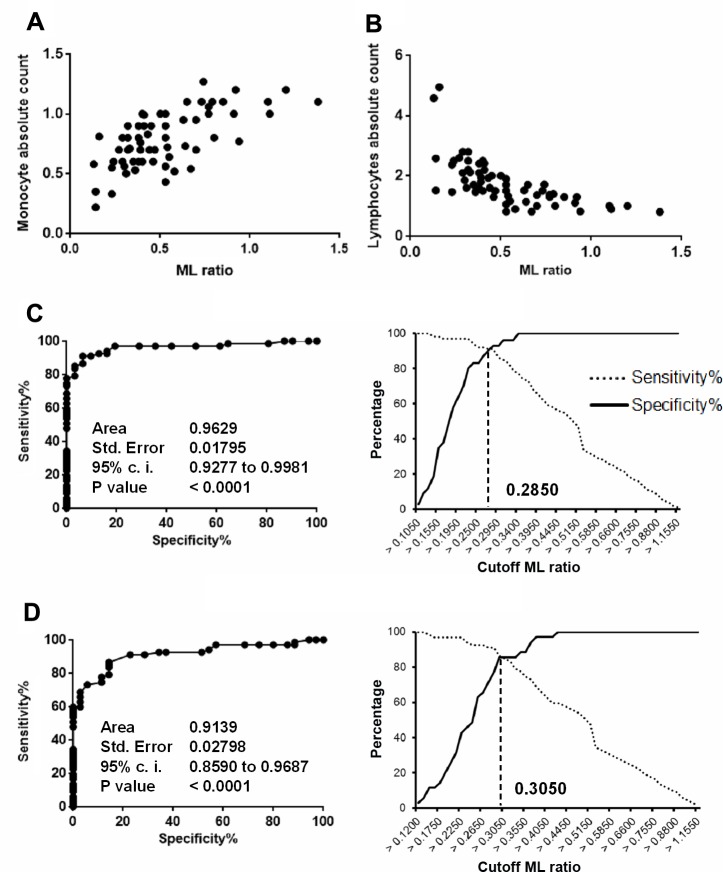
Correlation between the ML ratio and absolute monocyte and lymphocyte counts. A) The potential association between the ML ratio and absolute monocyte count, were analyzed by Spearman rank correlation test. Data shown are the values of the individual subjects. B) The potential association between the ML ratio and absolute lymphocyte count were analyzed by Spearman rank correlation test. Data shown are the values of the individual subjects. C) Receiver operating characteristic (ROC) curve for the ML ratio. The solid line shows the result for the value of ML ratio comparing active TB *vs* HD. D) The solid line shows the result for the value of ML ratio comparing active TB patients with LTBI subjects.

**Fig 3:**
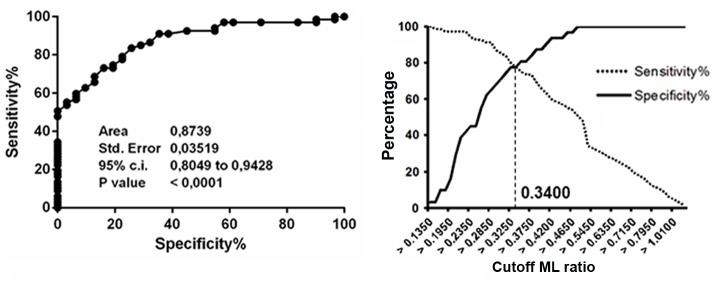
Receiver operating characteristic (ROC) curve for the ML ratio index. The solid line shows the result for the ML ratio value comparing patients with active TB disease to cured TB patients.

We then evaluated whether the increased ML ratio found in patients with active TB disease was dependent on changes in the numbers of lymphocytes, monocytes or both. As shown in **Table 2**and **Fig 1B**, the higher ML ratio found in patients with active TB disease was consistent with the associated significantly higher absolute monocyte counts (Median: 0.80, IQR:0.60–1.00) but slightly (and not significant) lower absolute lymphocyte counts (Median: 1.65, IQR: 1.30–2.03), compared to HD, and cured TB patients reaching the statistical significance when compared with LTBI subjects with a *p*<0.0001 (**Fig 1C**). There was a significant correlation between the ML ratio and absolute monocyte (R^2^: 0.4, r: 0.6, *p*<0.0001) and lymphocyte (R^2^: 0.6, r: -0.75, *p*<0.0001) counts, indicating that both the monocyte and lymphocyte counts contribute to the altered ML ratio (**Fig 2A and 2B**). Moreover, we analysed whether the predictive value of ML ratio was due to the frequency of monocytes count. Therefore we used the monocyte count for the negative binomial regression modeling considering the data from active TB patients included in the study. Monocyte count confirmed a slight association with active TB disease (Monocyte count IRR = 1.005 95% CI 1.0046, 1.0053, with a significance >99%). To further assess the accuracy of the ML ratio to discriminate between patients with active TB disease and other tested groups, ROC curves and cross-over plots were performed. As shown in **Fig 2C** the ML ratio well distinguishes patients with active TB from HD (AUC: 0.96, *p*<0.0001, cutoff: >0.28, 91.04% and 93.55%, of sensitivity and specificity, respectively), but was less robust to differentiate active TB patients from LTBI subjects (AUC: 0.91, p<0.0001, cutoff: > 0.30,85.07% and 85.71 of sensitivity and specificity, respectively, Fig 2D) and cured TB patients (AUC: 0.87, p<0.0001; cutoff: >0.34, 77.61% and 77.42% of sensitivity and specificity, respectively)(**Fig 3**).

### Phenotype of circulating monocytes during mycobacterial infection

**Fig 4:**
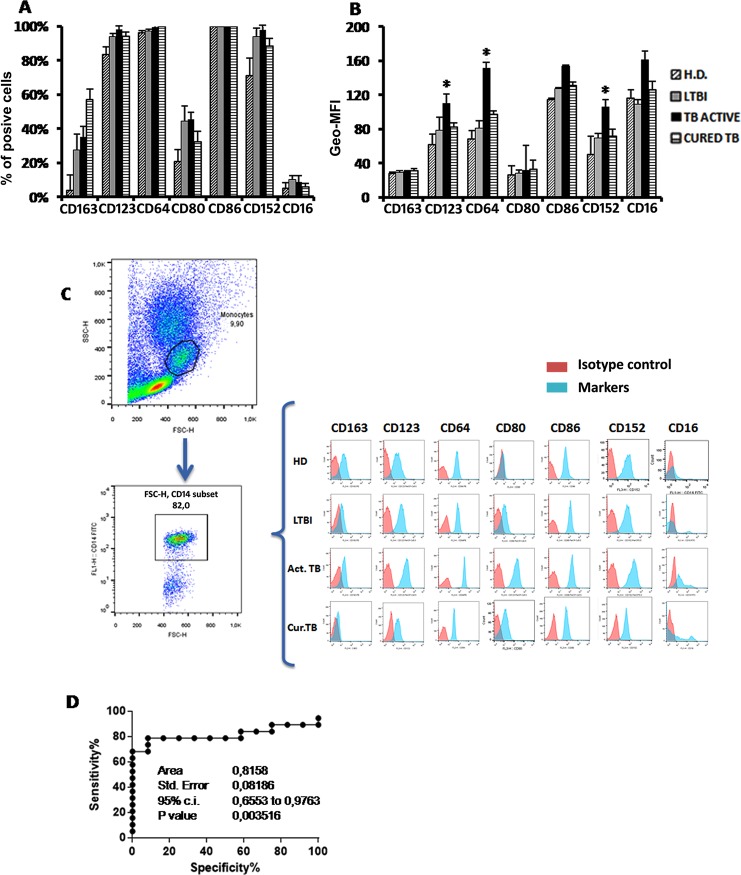
Surface molecules expression on circulating monocytes of patients with active TB disease, LTBI subjects, cured TB patients and HD. A) Cumulative data of the percentage expressed as median of surface expression and IQR. B) Geometric mean fluorescence intensity (geo-mean) of different surface molecules on monocytes and S.E. C) Representative FACS analysis of surface markers expression on circulating monocytes of one representative subject from each cohort group. D) Receiver operating characteristic (ROC) curve for the MFI value of CD64. The solid line shows the result for the value of comparing active TB *vs* LTBI.

We evaluated if the expression of certain activation molecules on the surface of monocytes may also represent a robust marker to distinguish between TB stages. As shown in **Fig 4A**, we did not find any significant difference in the percentage of circulating monocytes expressing any of the aforementioned surface molecules (see Materials and Methods) in different subject cohorts. Mean Fluorescent Intensity (MFI) was then measured to quantify surface molecule expression.

As shown in **Fig 4B**, the intensity of expression of three surface markers CD64, CD123 and CD152 was significantly higher in circulating monocytes from patients with active TB disease as compared to HD, and the intensity of expression of two of these markers (CD123 and CD152) was also significantly higher than in cured TB patients. **Fig 4C** shows primary cytometry histograms of one representative subject in each cohort group. However, and most interestingly, the intensity of expression of only one marker, CD64, on circulating monocytes allowed to discriminate between active TB patients and LTBI subjects (AUC value = 0.81, *p* = 0.003; **Fig 4D**).

In an attempt to investigate the clinical usefulness of CD64 expression on monocytes for discriminating active TB patients and LTBI subjects, we examined whether a metric incorporating the ML ratio to the CD64 MFI would generate an improved performance, because both values are increased in patients with active TB disease.

**Fig 5:**
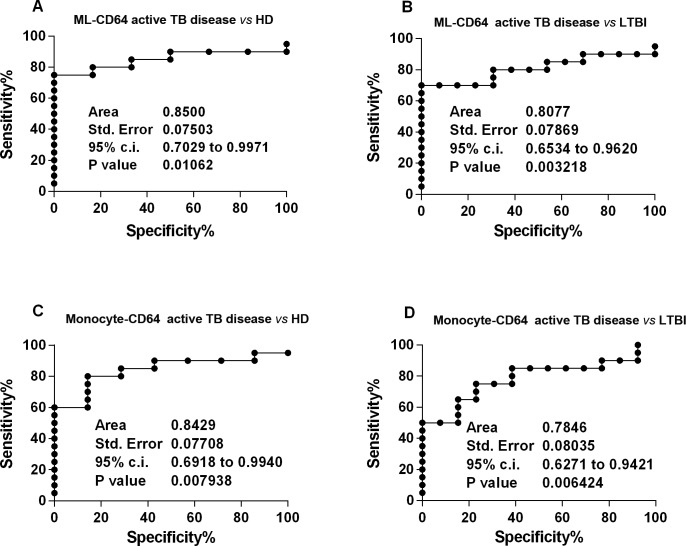
Receiver operating characteristic (ROC) curves for the ML-CD64 index and the Monocyte-CD64 index. A) The solid line shows the result for the ML-CD64 index value comparing patients with active TB disease to HD. B) The solid line shows the result for the ML-CD64 index value comparing patients with active TB disease to LTBI subjects. C) The solid line shows the result for the monocyte-CD64 index value comparing patients with active TB disease to HD. D) The solid line shows the result for themonocyte-CD64 index value comparing patients with active TB disease to LTBI subjects.

**Fig 6:**
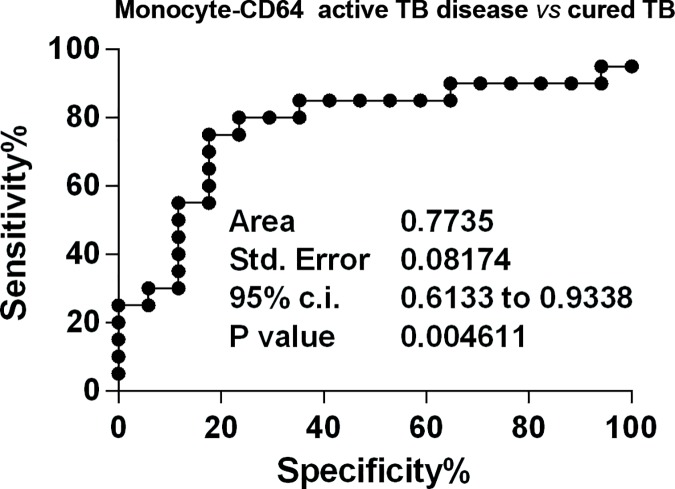
Receiver operating characteristic (ROC) curve for the monocyte-CD64 index. The solid line shows the result for the monocyte-CD64 value comparing patients with active TB disease to cured TB patients.

Therefore, we introduced a number of indices formulated by the ML ratio, or the absolute monocyte count, for the MFI of CD64^+^monocytes. We calculated these indices multiplying the value of geometric MFI of CD64 surface marker for the monocyte to lymphocyte ratio or for the absolute monocyte count, respectively. From here on, we referred to these indices as ML-CD64 and Monocyte-64, respectively. As shown in **Fig 5A and 5B**, the ML-CD64 index significantly discriminated between patients with active TB disease and HD (AUC = 0.850, *p* = 0.011) and LTBI subjects (AUC = 0,80, *p* = 0.003), while the Monocyte-CD64 index significantly discriminated patients with active TB disease from HD (AUC = 0.84, *p* = 0.007) **Fig 5C**, LTBI subjects (AUC = 0.78, *p* = 0.006) **Fig 5D** and cured TB patients (AUC = 0.77, *p* = 0.005) **Fig 6**.

Overall, these data show that the CD64-based indices display good accuracy in distinguishing between patients with active TB disease and HD.

## Discussion

Understanding the immune responses that underlies protection from infection or progression to disease is important to allow the development of diagnostic tools for the efficient prevention and management of TB [7].

Monocytes play a pivotal role as cellular component of the innate immune response and represent a link to the activation and modulation of the adaptive immune response due to their role as antigen presenting cells. Therefore, all the factors that could perturb the functions of monocytes, may potentially affect an individual response in the course of infections or autoimmune diseases or tumors.

Because CD4 T cells and monocytes/macrophages were previously shown to be major effector cells in protecting the host against Mtb infection [21], we investigated whether there were any differences in the monocyte compartment between patients with active TB disease and LTBI subjects, measuring their absolute number and phenotype and the consequent effect of their number on the M/L ratio.

In the present study we showed that patients with active TB disease had a very high ML ratio, as compared to both HD and LTBI subjects, as well as cured TB patients, suggesting that the ML ratio is changed after anti-TB therapy and could be used a tool to evaluate treatment success. In active TB patients, the ML ratio was significantly correlated with increased monocyte counts and lower lymphocyte counts, indicating that both the monocyte count and the lymphocyte count contribute to the altered ML ratio. Moreover, ROC curves and cross-over plots showed that the ML ratio could contribute to distinguish patients with active TB from HD, but was of poor value to differentiate active TB patients from LTBI subjects and cured TB patients.

ML ratio has been investigated in different context of infectious and non-infectious diseases. Recent studies have demonstrated that hematopoiesis may be perturbed by mycobacterial infection [22] or the pathogen may infect bone marrow mesenchymal stem cells [23]. In humans and in mice [24, 25], it has been demonstrated that subsets of hematopoietic stem cells have distinct biases in the ratio of myeloid and lymphoid cells they give rise to [26, 27].

Fletcher et al., have examined gene expression in 10-week-old BCG-vaccinated HIV-uninfected infants using whole-transcriptome micro arrays and have suggested that activation/quiescence of hematopoietic stem cell compartment was more prevalent among infants who developed TB respect to the infants that did not developed the disease [16]. From these data, the authors conclude that the transcriptomic may reflect leucocyte subset frequencies, suggesting that the quantity of myeloid and lymphoid transcripts in peripheral blood could be considered as a marker of the frequency of leucocyte subsets.

Naranbhai et al, reported that the ratio of ML in HIV-infected South African adults prior to combination antiretroviral therapy initiation (cART), was predictive of TB disease during the subsequent five years on cART [17]. Finally, and very recently, the same authors have demonstrated that increase of the ML ratio is associated with change of gene transcription in monocytes that may be correlated with their impaired anti-mycobacterial profiles, speculating on the association of this finding with pathophysiologic conditions [28]. Additionally, alteration in monocyte functions may alter crosstalk with lymphocytes and adaptive immune responses [29, 21], hence changes in monocyte functions alone may have a role in the detrimental immune response.

We have also investigated on the expression of circulating monocytes surface markers to identify whether a particular phenotype could be associated with the state of Mtb infection/disease. In fact, M1 macrophages are usually correlated with the resistance against intracellular pathogens such as *L*. *monocytogenes* and *S*. *typhimurium* [30, 31], as well as with the early phases of infection with bacteria such as *M*. *tuberculosis* [32, 24], *M*. *ulcerans*, and *M*. *avium* [33,34]. The excessive inflammation due to the uncontrolled M1 proliferation and activation is associated with acute bacterial infections and sepsis [35]. The switch of macrophages from M1 to M2 observed during the transition from acute to chronic infection, may provide protection against overwhelming uncontrolled inflammation; however, this switch could favor pathogens that have the ability to evade the immune response of the host interfering with M1-associated killing [33, 36–39].

Recently a modulation of M1-related, but not of M2-related genes evaluated by transcriptional profiles of patients with active TB has been shown. Similar results were obtained in infants vaccinated with bacillus Calmette–Guerin [40–42].

In the present study, we didn’t find any clear M1 or M2 polarization of circulating monocytes. Differently, we found that CD64, CD123 and CD152 were over expressed in patients with active TB disease respect to HD group. Moreover, expression of CD64, measured as MFI, allowed the discrimination between active TB patients and LTBI subjects. Additionally, CD64-expressing monocyte-based indices gave better performance in discrimination between patients with active TB disease and LTBI subjects.

The meaning of the over expression of these molecules could be partially explained with their role played during the different TB stages. CD64is able to induce phagocytosis, respiratory burst and antibody-dependent cell-mediated cytotoxicity in granulocytes [43]. CD64 protein expression is increased in monocytes from TB patients and as well as the CD64 expression at the RNA and cell surface protein level [43]. CD64 is induced by type-I and type-IIIFN [43, 44], suggesting that the modulation of expression of certain surface markers on human monocytes might reflect differential cytokine production and mark different stages of Mtb infection/disease.

In conclusion, in this pilot study we found a correlation between the absolute number and phenotype of monocytes and the different TB stages. The comprehension of the mechanisms involved in the change of this cellular population should be confirmed in a larger cohort of patients.

## Supporting information

S1 File File contains full data of lymphocyte and monocyte count for each subject included in the study. Data supporting information La Manna et al.xlsx.
(XLSX)
